# Microbubbles bound to drug-eluting beads enable ultrasound imaging and enhanced delivery of therapeutics

**DOI:** 10.1038/s41598-024-71831-3

**Published:** 2024-09-09

**Authors:** Joshua Owen, Ayele H. Negussie, Scott R. Burks, Jose Delgado, Andrew S. Mikhail, Jocelyne Rivera, William F. Pritchard, John W. Karanian, Eleanor Stride, Joseph A. Frank, Bradford J. Wood

**Affiliations:** 1 Center for Interventional Oncology, Radiology and Imaging Sciences, Clinical Center, National Institutes of Health, Bethesda, MD USA; 2 Frank Laboratory, Radiology and Imaging Sciences, Clinical Center, National Institute of Biomedical Imaging and Bioengineering, National Institutes of Health, Bethesda, MD USA; 3 Institute of Biomedical Engineering, Department of Engineering Science, University of Oxford, Oxford, UK

**Keywords:** Translational research, Biomedical engineering

## Abstract

Transarterial chemoembolization (TACE) is an image-guided minimally invasive treatment for liver cancer which involves delivery of chemotherapy and embolic material into tumor-supplying arteries to block blood flow to a liver tumor and to deliver chemotherapy directly to the tumor. However, the released drug diffuses only less than a millimeter away from the beads. To enhance the efficacy of TACE, the development of microbubbles electrostatically bound to the surface of drug-eluting beads loaded with different amounts of doxorubicin (0–37.5 mg of Dox/mL of beads) is reported. Up to 400 microbubbles were bound to Dox-loaded beads (70–150 microns). This facilitated ultrasound imaging of the beads and increased the release rate of Dox upon exposure to high intensity focused ultrasound (HIFU). Furthermore, ultrasound exposure (1 MPa peak negative pressure) increased the distance at which Dox could be detected from beads embedded in a tissue-mimicking phantom, compared with a no ultrasound control.

## Introduction

Hepatocellular carcinoma (HCC) is the most common primary liver cancer and the 5th leading cause of cancer mortality in the United States^1,2^ HCC typically occurs on the background of chronic inflammation resulting from human viral hepatitis, excessive alcohol consumption, or nonalcoholic fatty liver disease associated with diabetes or obesity^3^. It is usually diagnosed at later stages of tumor development after it is no longer possible to treat via transplant or surgical resection. For such patients, loco-regional therapies (LRT) such as transarterial chemoembolization (TACE), radioembolization, radiofrequency, microwave and cryo ablation, may be employed as palliative options^4^.

TACE involves arterial injection of a chemotherapeutic and embolization of the vessel using solid particles, gelatin, or oil emulsion for the combined impact of ischemia and cytotoxicity^5^ or use of an emulsified chemotherapeutic in ethiodized oil administered transarterially with or without a solid embolic^6^, which is now termed as conventional TACE (cTACE). The theory is that disruption of blood flow to tumors in combination with direct delivery of a cytotoxic agent will have a synergistic tumoricidal effect. To standardize this technique and make it more reproducible, embolic microspheres loaded with cytotoxic agents, called drug-eluting beads (DEB), were developed^4,7^.

The use of microspheres to enhance drug delivery is based on the original concept that arresting blood flow after drug delivery can prevent washout and reduce systemic drug exposure, thereby reducing side effects^8,9^. TACE with DEB results in a slower and more continuous deposition of drug in tumors and reduced systemic exposure compared to cTACE^7,10^. In a retrospective analysis, when patients were stratified according to tumor morphology—infiltrative vs. nodular disease—patients with nodular disease had significantly prolonged survival when receiving DEB-TACE as compared to patients receiving cTACE, although potential selection bias exists^11^. There are clinical trials underway combining locoregional therapies such as TACE with immunotherapy^12^. Many types of microspheres are also available on the market with a potentially large pool of drugs to choose from for delivery via DEB-TACE.

Drug loading is normally performed at the point of use by mixing a drug solution with DEB^13–15^. Loading of Dox and similar drugs into DEB occurs via interaction with negatively charged sulfonate or carboxylate moieties within the microsphere^13,16,17^ while drug elution occurs by the action of physiological counter ions^18^. However, the rate of drug diffusion and transport in tumors is influenced by the physicochemical characteristics of the drug including molecular size, charge, and solubility as well as the extent and location of cell uptake and binding^19–22^. Drug transport is primarily governed by diffusion as convective transport is reduced by the blockage of blood vessels resulting from TACE.

DEB-TACE suffers from a lack of understanding of the relative contributions of drug versus ischemia towards treatment efficacy. Comparisons of studies that performed embolization with bland microspheres to those that performed DEB-TACE reveal no consensus regarding which technique is clinically superior^4,23,24^. For instance, there is no significant difference in overall survival (OS) between patients treated with bland or drug-loaded DEB, although perhaps less side effects with DEB-TACE. One issue which may limit efficacy is the poor penetration of drug from DEB into the surrounding tumor. This process is mainly driven by diffusion of drug (e.g., Dox) across the blood vessel wall into the tumor extravascular space*.* Studies that have evaluated the temporospatial distribution of drugs in tumors found that Dox remains only within a 600 µm radius of the microspheres^25–28^.

**Fig. 1:**
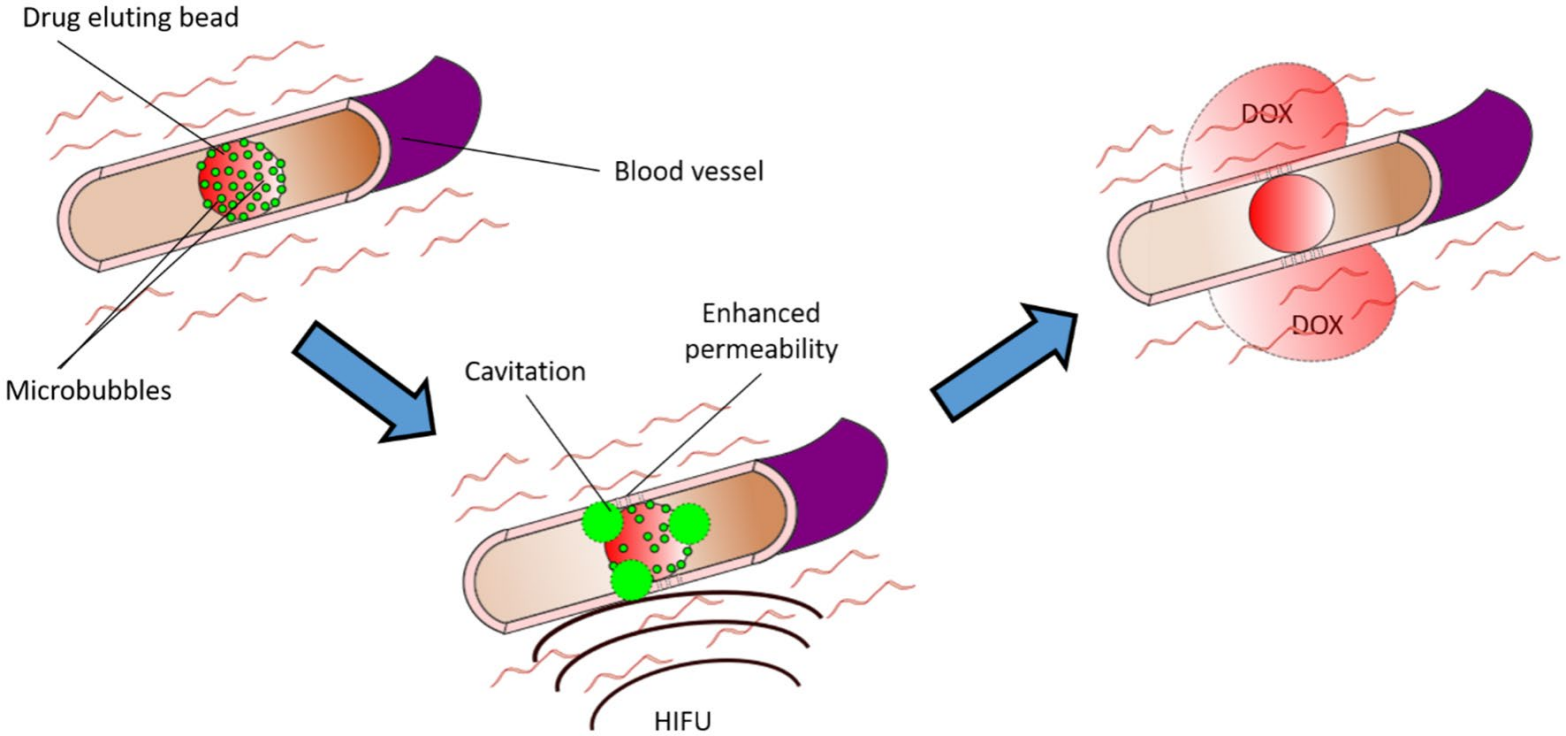
Schematic of delivery mechanism of drug-eluting beads with microbubbles (MB-DEB). The MB-DEB block the blood vessel followed by application of high intensity focused ultrasound (HIFU) to cause cavitation of the microbubbles which enhances the permeability of the surrounding blood vessel resulting in improved distribution of doxorubicin (Dox) into the surrounding tumor tissue.

One method to enhance the transport of doxorubicin into the surrounding tissue could be to exploit the mechanical effects produced by cavitation, which can be initiated using ultrasound and microbubbles (MB)^29^. Lipid-coated gas MB with diameters in the range 1–10 µm are currently used clinically as contrast agents to highlight blood flow in ultrasound imaging^30^. However, under high intensity focused ultrasound (HIFU) MB undergo large amplitude volume oscillations. This process can both permeabilize cell membranes and drive therapeutic molecules out of the vasculature into the surrounding tissue through a variety of mechanisms including microstreaming^31^. This process has been used to deliver therapeutic agents of various sizes to tumor tissue^32–34^. For example, Price et al*.* used MB cavitation to create microvessel ruptures large enough to permit the extravasation of red blood cells whilst limiting tissue damage to the rupture site itself. The hypothesized procedure would be to add MB onto DEB just before delivery to patients using standard clinical operating procedures requiring minimal additional time, equipment, cost, or ergonomic disruption. After the microbubble loaded DEB have been delivered, HIFU or ultrasound could be applied to cavitate the MB, permeabilizing the blood-vessel wall and potentially enhancing Dox penetration into the surrounding tumor tissue (Fig. 1).

As a secondary effect, MB attached to the surface of DEB could also be used to enable visualization of the TACE procedure using ultrasound imaging. DEB platforms that can be imaged with x-rays, i.e., fluoroscopy or computed tomography, have been developed by covalently attaching different radio-opacifier-containing moieties^35–38^. The first-in-human use of an iodinated microsphere which provided real-time feedback and geographic localization of treatment was reported in 2016^39^ with many others under development^38^. Spectral imaging techniques in computed tomography (CT) can be used to differentiate radiopacifiers such as iodine, bismuth or tantalum such that location or quantity of DEB with different radiopacifiers and different therapeutic drugs could be distinguished^40^. The addition of ultrasound contrast agents to the DEB could enable a low-cost imaging method to quickly confirm the location, or in theory, spatial dosimetry of DEB with MB. Ultrasound imaging could also provide a method to confirm cavitation had taken place after application of HIFU through the signal from the bubbles. Bubbles could be detected by ultrasound before cavitation and this signal would no longer be detectable after cavitation.

The aim of this study was to investigate the feasibility of this approach. The study included synthesis and characterization of the microbubbles, development of the binding of MB to DEB and characterization of the combined materials, demonstration of enhanced drug release from MB-DEB with cavitation and enhanced drug penetration into a vessel phantom, and finally imageability of the beads under diagnostic ultrasound.

## Materials and methods

### Materials

Distearoyl phosphatidylcholine (DSPC), 1,2-distearoyl-3-trimethylammoniumpropane (TAP) and 1,2-distearoyl-sn-glycero-3-phosphoethanolamine-N-(7-nitro-2-1,3-benzoxadiazol-4-yl) (ammonium salt) (NBD-DSPE) were purchased from Avanti polar lipids. Saline, Chloroform, PEG-40-stearate, glycerol, and propylene glycol were purchased from Sigma Aldrich. Agarose and alginic acid were purchased from ThermoFisher Scientific. 3D printing material, thick black polyvinyl alcohol (PVA), for 3D printing was purchased from 3D Herndon. Polydimethylsiloxane (PDMS) and the curing agent (Sylgard 184) were purchased from Dow Inc. LC Bead M1 Beads (70–150 µm) (M1) and LUMI M0 Beads (40–90 μm) (LUMI) (Biocompatibles Ltd, now Boston Scientific), and loaded with doxorubicin as previously reported^41^.

### Microbubble manufacture

MB were produced as previously described by Christiansen et al.^42^. Briefly, PEG-40-stearate, DSPC and TAP in a molar ratio of 5:4:1, respectively, were sonicated in an aqueous dispersion to produce cationic-MB under a positive pressure of sulfur hexafluoride. To demonstrate that a positive charge was important for the adsorption onto drug eluting beads, neutral-MB were also produced following the same process above without using TAP. To make fluorescent microbubbles, NBD-DSPE (1%) was added to the MB formulation and the same procedure was followed.

### Microbubble size analysis

MB were assessed for size via optical microscopy (AxioImager M1, Zeiss, Thornwood, NY). MB, 10 μl, were added to a hemocytometer and multiple images (n = 20) were taken at 10× magnification. These images were then analyzed in MATLAB (Mathworks, Inc., Natick, MA) using a previously published technique to assess microbubble concentration^43^.

### Charge measurement

The zeta potential of neutral-MB, cationic-MB and M1 beads were measured via a Zetasizer Nano (Malvern, Westborough, MA). Approximately 1 × 10^7^ MBs were dispersed in 1 mM KCl at pH 7.4 and their charges were measured.

### Binding of microbubbles to beads

LCM1 and LUMI M0 are both anionic microspheres based on polyvinyl alcohol. LCM1 particles range in size from 70 to 150 μm while LUMI M0 ranges from 40 to 90 μm. LUMI M0 has iodine covalently bound to the beads rendering them imageable on x-ray or CT. Both LC and LUMI beads are cleared for clinical use. Both can be loaded with doxorubicin to generate drug eluting beads (DEB) for transarterial embolization, although regulatory approval for drug loading varies by jurisdiction. To bind microbubbles to M1 beads (MB-M1), microbubbles were concentrated by centrifugation (1000 RCF, 10 min) in water (1 mL). Concentrated MB (50 µl) were then added to deionized water (200 µl) followed by the addition of M1 beads (120 µl) to the microbubble solution. The M1 would settle to the bottom of the vessel and could be separated from non-bound MB. The solution could also be centrifuged (200 RCF, 1 min) to separate MB-M1 from unbound MB more rapidly.

### Quantification of microbubble binding

M1 were loaded at 0%, 25%, 50%, 75% and 100% Dox, where 100% Dox loading is a clinical loading concentration of 37.5 mg Dox/ml of DEB. MB were then added to the Dox-loaded M1 beads via the same method outlined above (MB-M1 DEB). The MB-M1 DEB were then analyzed via microscopy, acquiring images of at least 10 MB-M1 DEB using a 20× objective upright microscope (AxioImager M1, Zeiss) equipped with a color CCD camera (AxioVision, Zeiss). The MB on the bead surface were counted manually in FIJI (National Institutes of Health, Bethesda, Maryland, USA) by light microscopy and via fluorescence signal. Z-stack images were acquired using a Zeiss LSM 710 confocal microscope system (Zeiss, Thornwood, NY) using an excitation of 467 nm and emission 539 nm for NBD for bubble detection and an excitation of 480 nm and emission of 590 nm for Dox. A z-stack of the MB-DEB was obtained starting in the center of the bead and taking images every 10 µm to the top of the bead (40–80 µm depending on DEB size) with a 20× objective. The number of MB per M1 were then estimated for the whole DEB surface and plotted for each percent Dox loading of M1.

### Release study

For the release study involving cavitation, the MB-M1 DEB had HIFU applied at room temperature in saline in an ultrasound transmissible PDMS device holding approximately 800 µl volume of MB-M1 DEBs. HIFU conditions were 1 MHz, 1 MPa, 30% duty cycle, 10 ms pulse length and a PRF of 100 Hz for 5 s. The MB-M1 DEB solution was then transferred to 15 ml of saline and placed in a rotating mixer (Ward’s^®^ Rotating Mixers, VWR, Bridgeport, NJ) and agitated with an upright rocking motion at 37 °C at 70 RPM based on a method reported by Negussie et al.^44^. This was designed to keep the saline well-mixed, acting as an infinite sink to optimize diffusion of dox from the bead and allow for comparison of release rates after different stimuli. The preparation was agitated and 200 µl samples were then taken from the sample at t = 0 and then every hour for 8 h and then at 24 and 48 h. An equivalent volume of saline, 200 µl, was then added after each sample was removed to maintain the total volume throughout the release study. The application of HIFU and transfer into the saline took approximately 1 h. As controls, MB-M1 DEB were exposed to the same temperature conditions and treatment environment but without HIFU exposure. The release study was performed for MB-M1 DEB loaded with 75% and 100% Dox. The assay for Dox took place via High Pressure Liquid Chromatography (HPLC) using a standard protocol from Negussie et al. using an LC1290 (Agilent, Santa Clara, CA, USA)^44^.

### Delivery study

**Fig. 2:**
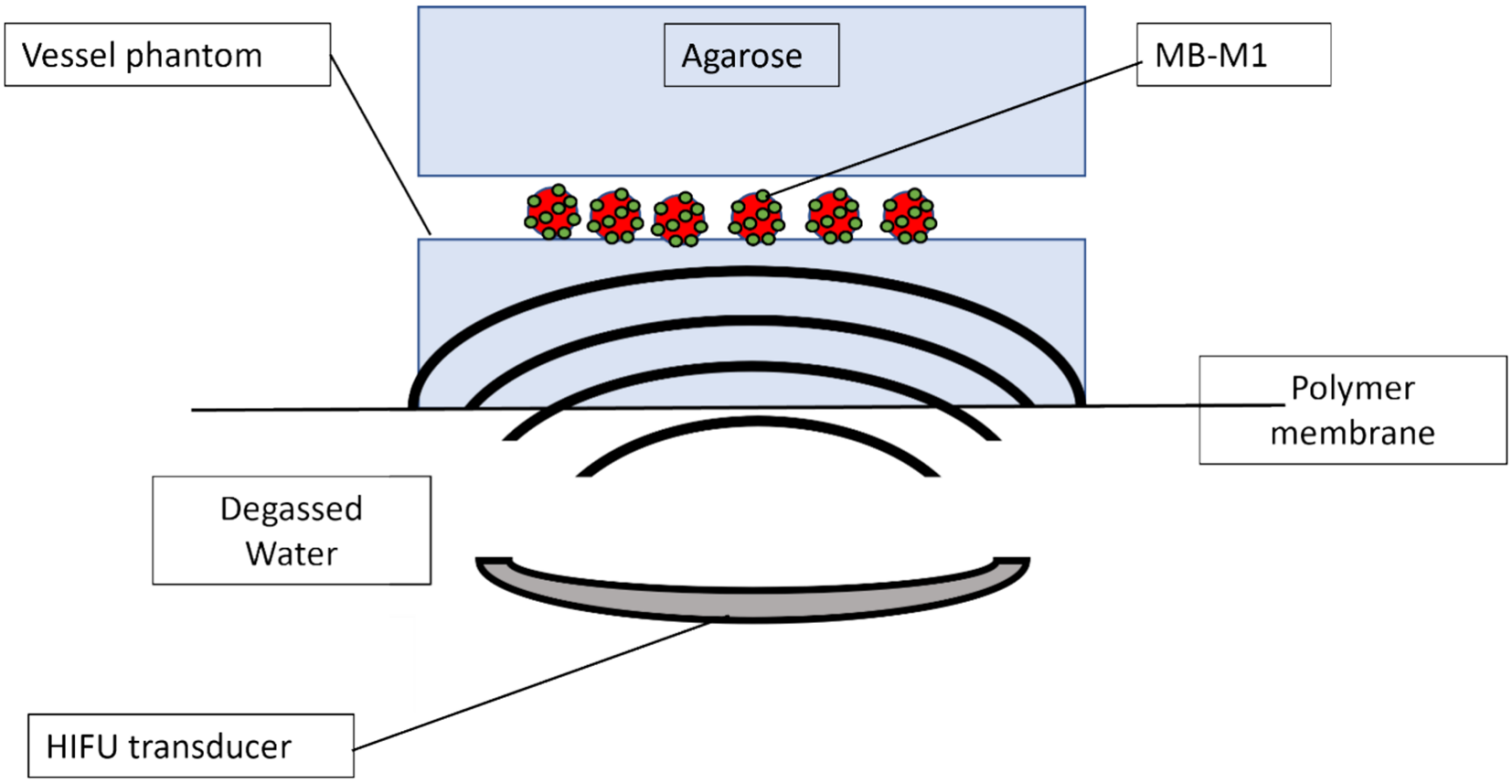
Schematic of setup for investigating Dox penetration into Agarose from microbubbles-drug eluting beads (MB-M1) with and without high intensity focused ultrasound (HIFU). Objects are not to scale.

Ultrasound drug release studies were performed in a 1% agar, 0.5% alginic acid vessel phantom as a tissue-mimicking material (TMM) with a tubular lumen to represent a vessel, prepared by pouring the TMM into a 3D-printed mold. The TMM was degassed during the process of setting in the mold. This produced a channel within the TMM with a diameter sufficiently large to be visualized via magnetic resonance imaging (MRI) (diameter = 3 mm). For reference, the hepatic artery and the right and left hepatic arteries are approximately 2–5 mm in diameter^45^. The ends of the phantom could also be sealed to contain the MB-M1. The vessel was targeted using MRI guided HIFU. The HIFU (FUS Instruments, Toronto, ON, Canada) transducer had a center frequency of 1.125 MHz, an aperture of 75 mm, 60 mm diameter with an F# 0.8 Radius of Curvature. calibrations for total power output were conducted using an acoustic radiation force balance (UPM-DT-50SP Ultrasound Power Meter, Ohmic Instruments Inc) with degassed water at 25 °C. A 3.0 T MRI system (Philips, Best, the Netherlands) was used and HIFU targets were planned off of T2-weighted images with an in-plane resolution of 0.1mm. The HIFU beam was registered in MR space using a phantom. HIFU was applied from below at 1 MHz, 1 MPa, 30% duty cycle, 10 ms pulse length and a PRF of 100 Hz for 5 s at set points approximately 0.2 mm apart along the length of the vessel (Fig. 2). Phantoms (n = 3 for each time point) were then sectioned in a plane perpendicular to the axis of the lumen at 25 min and 100 min after HIFU application. The sectioned agar was then examined via fluorescence microscopy (Imager M1 Axio Fluorescence Microscope, Zeiss) at an excitation peak of 480 nm and an emission of 590 nm and a stitched figure of the vessel wall was prepared. This vessel section was then analyzed via radial profiles in FIJI and penetration depth of the Dox was averaged over 3 vessel sections and plotted using Python (Centrum Wiskunde & Informatica, Amsterdam, the Netherlands). This was then compared to MB-M1 DEB without HIFU. MB-M1 DEB loaded with 75% and 100% Dox were examined to compare Dox quantity vs MB quantity for drug release.

### Ultrasound imaging

The same phantoms used for examining Dox delivery were also used for examining the imaging capability of MB-LUMI under ultrasound and CT. LUMI were selected to allow for confirmation between the two imaging modalities. With the vessel phantom filled with saline, MB, MB-LUMI, or LUMI were injected into the lumen under ultrasound imaging (Sonosite Ultrasound, Fujifilm Sonosite, Inc, Bothell, WA) with an L25 x Linear probe, to ensure correct positioning of the needle. Flow was then stopped allowing beads to sediment or MB to rise. A separate phantom was used for each to prevent contamination. After injection of the sample, US B-mode videos were recorded for one minute after injection to allow sedimentation. Snapshots were then taken after sedimentation and an area of interest selected for pixel analysis. The average pixel intensity within this region was then measured. After an ultrasound video had been obtained, cone beam computed tomography images were acquired (Allura Xper FD20 X-ray system; Philips) at 80 kVp with a spatial resolution of 0.656 mm. The CT images were then examined using the Mimics Research software package (version 20.0; Materialise, Leuven, Belgium) to determine MB-LUMI location in the vessel.

## Results and discussion

### Microbubbles

**Fig. 3:**
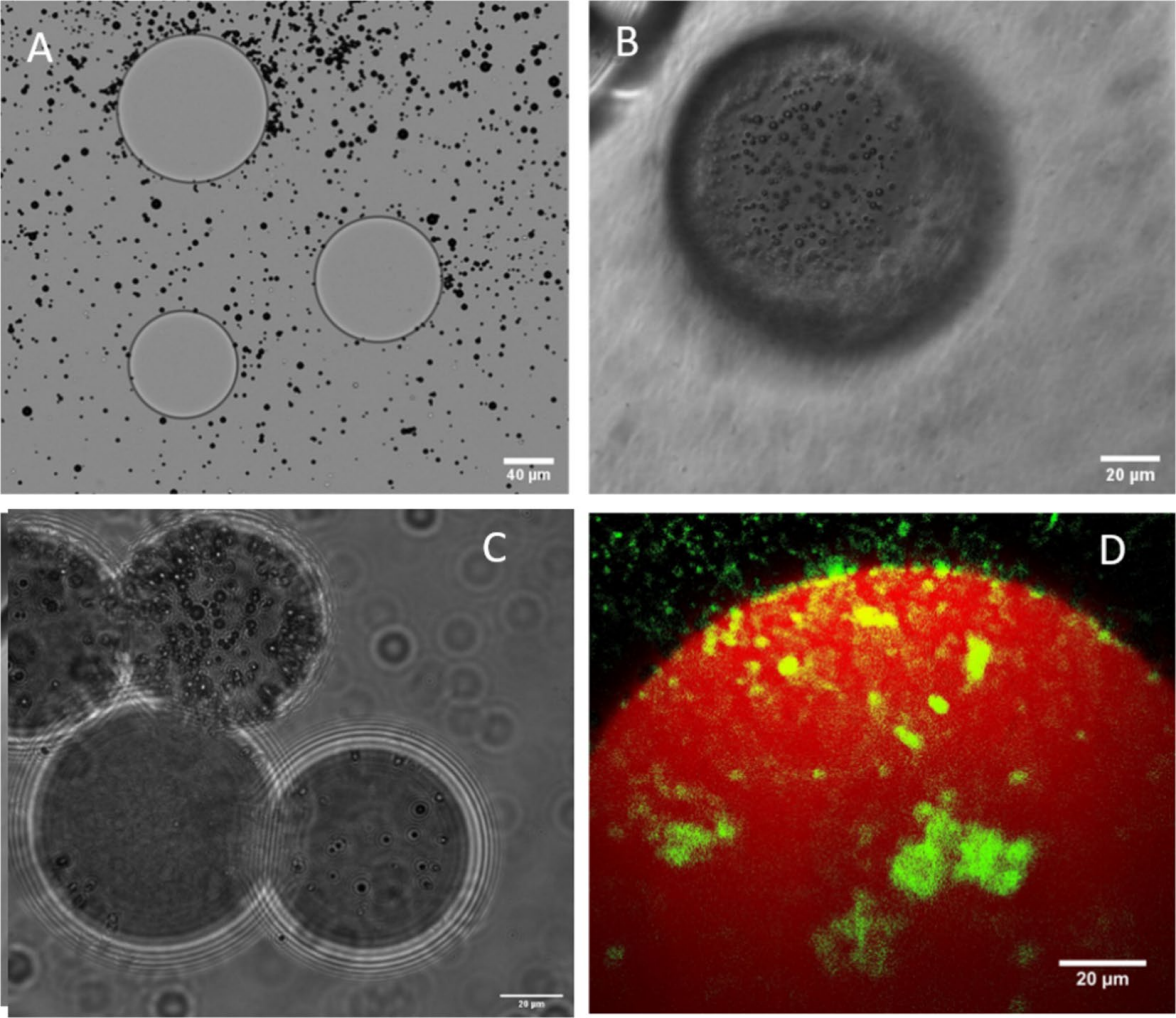
Microbubble (MB) adsorption to beads. (**A**) Unloaded M1 with neutral MB shown in the midplane, (**B**) unloaded M1 bead with cationic MB shown above the midplane after centrifugation, (**C**) unloaded LUMI with cationic MB shown in the midplane after centrifugation and (**D**) a z-stack of a 100% doxorubicin loaded M1 DEB (red) with fluorescent cationic MB (green).

Cationic and neutral MB were made at a concentration of 1 × 10^9^ MB/mL and a size range between 2 and 10 µm (Supplementary material). The microbubbles were centrifuged twice at 1000 g for 10 min to remove all nanoscale objects created during the sonication process. Charge analysis via zeta potential showed neutral microbubbles had no charge whereas cationic microbubbles had a zeta potential of 31 mV (Supplementary material). This charge was sufficient to electrostatically bind the MB to anionic M1 as shown in Fig. 3. The dye (DSPE-NBD) was also incorporated into the cationic MB to visualize MB under fluorescence microscopy to improve observation of MB on Dox loaded M1.

### MB-DEB association

To bind MB to DEB, the electrostatic charge of the DEB was exploited. This is currently used for loading Dox but was also used to bind cationic MB. As shown in Fig. 3A neutral MB and unloaded M1 showed minimal signs of MB adsorption to the M1 surface. When the experiment was repeated with cationic MB large quantities of MB were associated to the unloaded M1 surface (Fig. 3B) and on the unloaded LUMI surface (Fig. 3C) after removing unbound microbubbles through centrifugation. This was repeated with cationic MB incorporating the fluorophore DSPE-NBD and Dox loaded M1 and viewed via confocal microscopy. As shown in Fig. 3D the green microbubbles are observed bound to the red M1 surface. As Dox loaded M1 have a reduced charge relative to unloaded M1 it is likely to have a lower attraction and as such bind fewer MBs. To determine any differences in binding efficiency, the number of MB bound to the DEB surface for different Dox loading was determined from microscopy images.

### Quantification of microbubble binding

**Fig. 4:**
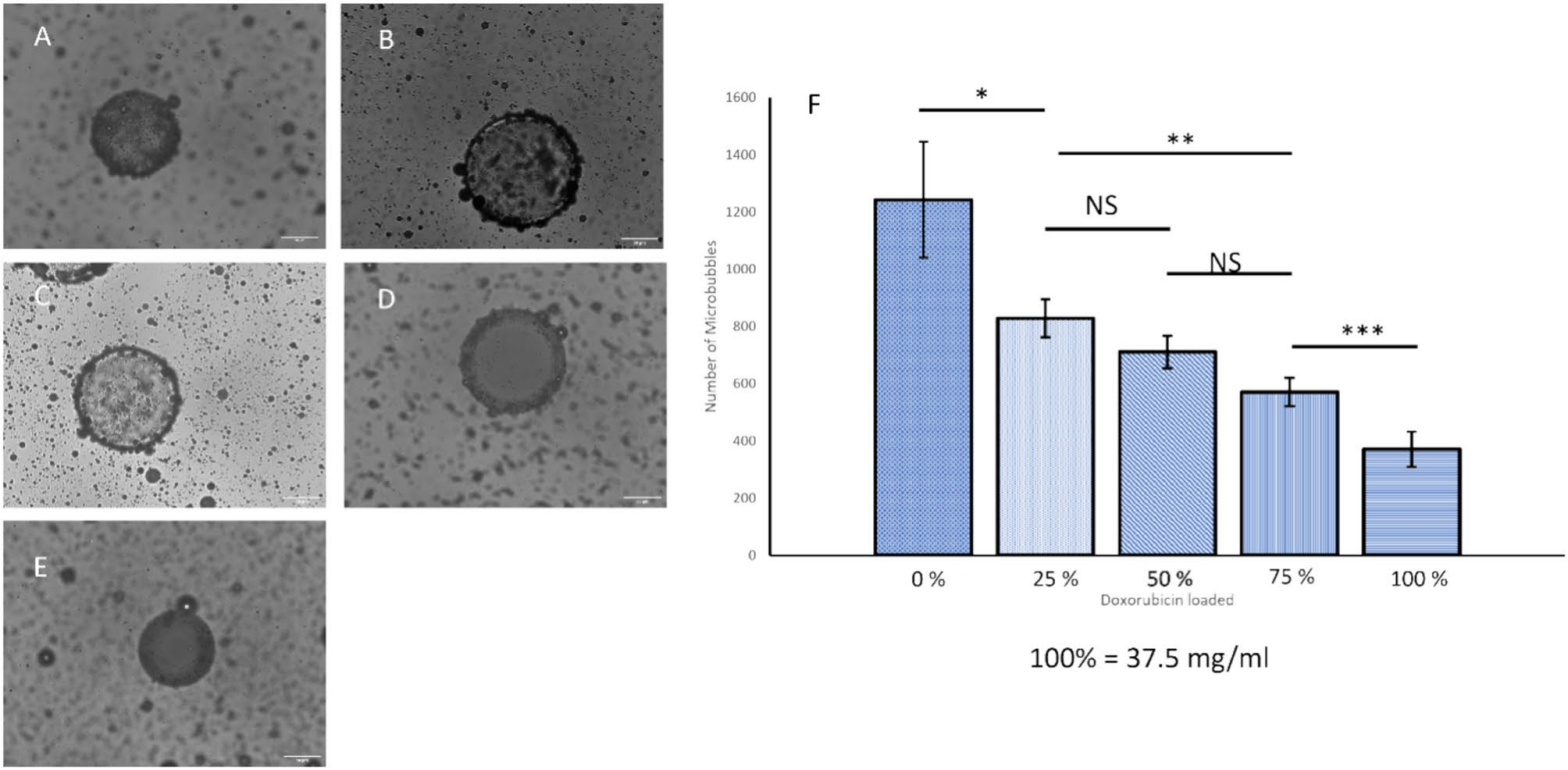
Microscopic quantification of microbubble binding to beads relative to quantity of Dox loaded up to 37.5 mg/ml (100%). (**A**) Image of microbubbles bound to M1 DEB with 0% Dox, (**B**) 25% Dox, (**C**) 50% Dox, (**D**) 75% Dox, (**E**) 100% Dox. (**F**) quantifying the number of microbubbles bound to beads with different concentration of Dox Loaded (n = 15 for each percentage dox loaded).

In order to quantify the impact of Dox loading on MB binding, the amount of Dox in the M1 DEB was varied, using 0%, 25%, 50%, 75% and 100% where 100% Dox loading is 37.5 mg Dox/mL, as this is loading commonly used in investigational and clinical practice^14,46^. As expected, Dox loading reduced the quantity of microbubbles bound to the DEB surface (Fig. 4A–E), ranging from approximately 1200 MB per M1 bead with no Dox to approximately 400 MB per M1 bead at 100% dox loading (Fig. 4F). This reduction provides strong evidence for the electrostatic binding of MB to beads. The method of attachment of the MB to the DEB also fits within the clinical operating procedures for use of Dox loaded DEB. To load Dox onto the DEB, the beads are added to the solution containing Dox and left for approximately 24 h after which they are ready to be injected into the patient. Attaching the MB would only require the manufacture of the MB which can be accomplished in under 5 min, in a similar manner to SonoVue©, followed by addition of the MB to the DEB vial and brief agitation and delivery to the patient. Thus, it represents only a minimal addition to existing clinical delivery procedures. However, further work is required to explore the impact of non-bound microbubbles. It is unlikely that non-bound microbubbles would substantially influence the delivery of the DEBs due to their relative size and density. Any unbound MB would enter the circulatory system and be removed by the lungs and liver in a matter of minutes. However, charged microbubbles in circulation could also result in some off target effects or potential immune response. At the site of delivery, cavitation of unbound MB could affect the beads with increased drug release or the vessel wall leading to increased permeability. This will need to be explored in further in vivo studies. Alternatively, the preparation could be centrifuged beforehand to remove the unbound bubbles, but this would add further steps to the clinical protocol. Alternatively, the amount of MB added to the DEB could be more rigorously controlled in order to minimize the amount of free MB.

However, to further explore the therapeutic efficacy of the MB-DEB constructs and determine whether the quantity of MB or the amount of Dox was the key factor in drug delivery and release, M1 DEB loaded with 75% or 100% Dox were studied further.

### Ultrasound imaging/CT of microbubble loaded LUMI

**Fig. 5:**
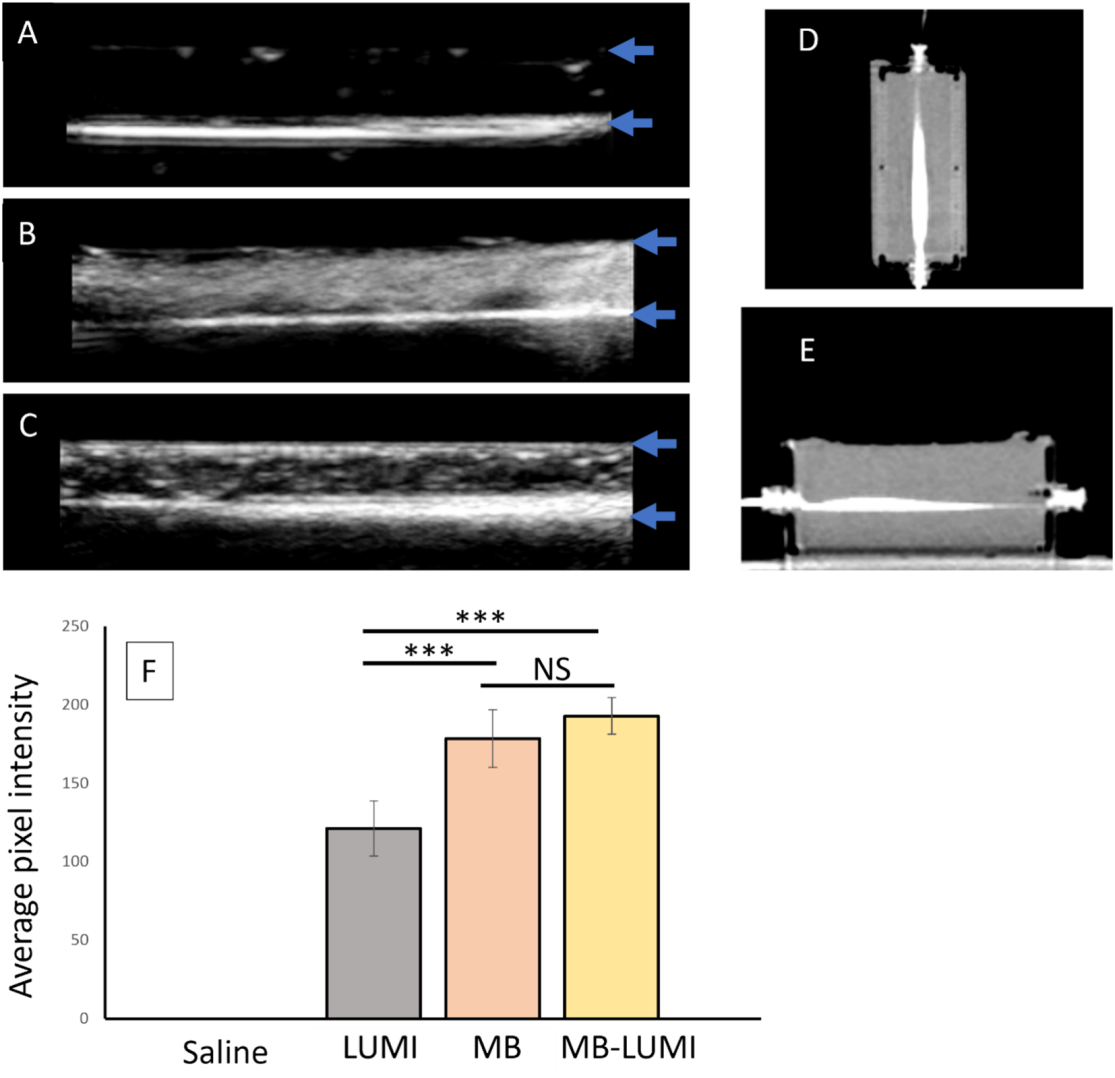
Imaging of LUMI-DEBs bound to microbubbles inside a tissue-mimicking phantom. Ultrasound images of (**A**) LUMI, (**B**) MB alone and (**C**) MB-LUMI. (**D**) CT images of LUMI bound to microbubbles along the horizontal plane and (**E**) the vertical plane showing correlation with the location of the beads in the ultrasound. (**F**) Pixel intensity analysis of ultrasound images of saline, LUMI, MB alone, and MB-LUMI. The vessel has a diameter of 3 mm. Blue arrows indicate lumen boundaries. For all graphs (n = 3).

To examine the imageability of MB-LUMI under ultrasound a channel was made from agar/alginate TMM and used to inject three groups, LUMI, MB, and MB-LUMI. The injection was observed under ultrasound and then the flow paused to acquire static images for analysis of signal intensity. Images of the LUMI are shown in Fig. 5A, MB in Fig. 5B and MB-LUMI in Fig. 5C. The signal intensity of the three groups was then analyzed (Fig. 5F) which showed no statistical difference between MB and MB-LUMI (p = 0.24) after a one-way Anova. However, both MB-LUMI (p = 0.0000) and MB (0.0000) had a higher pixel intensity than LUMI alone indicating potential use for ultrasound imaging and contrast enhanced ultrasound imaging to guide the TACE procedure. MB-LUMI were also concentrated at the bottom of the vessel whereas MBs alone were dispersed throughout, reflecting the density of the iodinated microbeads. The MB-LUMI should also appear in the contrast mode of ultrasound imaging although this was not examined. The exact change in acoustic response of the MB after having been bound to MB-LUMI relative to free MB also requires further investigation. Also, the destruction of microbubbles over time via ultrasound imaging requires quantification before clinical use. The presence of unbound MB can also be observed above MB-LUMI in the vessel. The MB-LUMI can be differentiated owing to the larger area of signal intensity along the base of the vessel however, this demonstrates that in future work, controlling or reducing the unbound MBs to levels where they do not interfere with or shadow MB-LUMI signal would need to be explored. As LUMI are visible on CT imaging, an image of the phantom was also acquired after injection of MB-LUMI clearly showing the injected beads (Fig. 5D and E). This then allows for cross-correlation of ultrasound and CT imaging. The ultrasound may also allow for measurements of signal intensity to determine if MB are still present on the MB-LUMI after infusion into the body. Changes in average pixel intensity after HIFU could potentially be used to assess the presence of MB and if HIFU caused any reduction in signal intensity. MB-LUMI may also be differentiated from other DEB, forming a component of imaging assessment of delivery of multiple DEB types imageable via CT and US. For future clinical use, the bubble bead should fit into current clinical procedures allowing for introduction of ultrasound imaging as well as CT to assess bead localization. Should cross correlation of CT and ultrasound show good agreement in future it may be possible to only use ultrasound for confirmation of delivery thus reducing the cost and radiation exposure of a procedure. However, this will only take place after years of clinical evidence.

### In vitro release studies

**Fig. 6:**
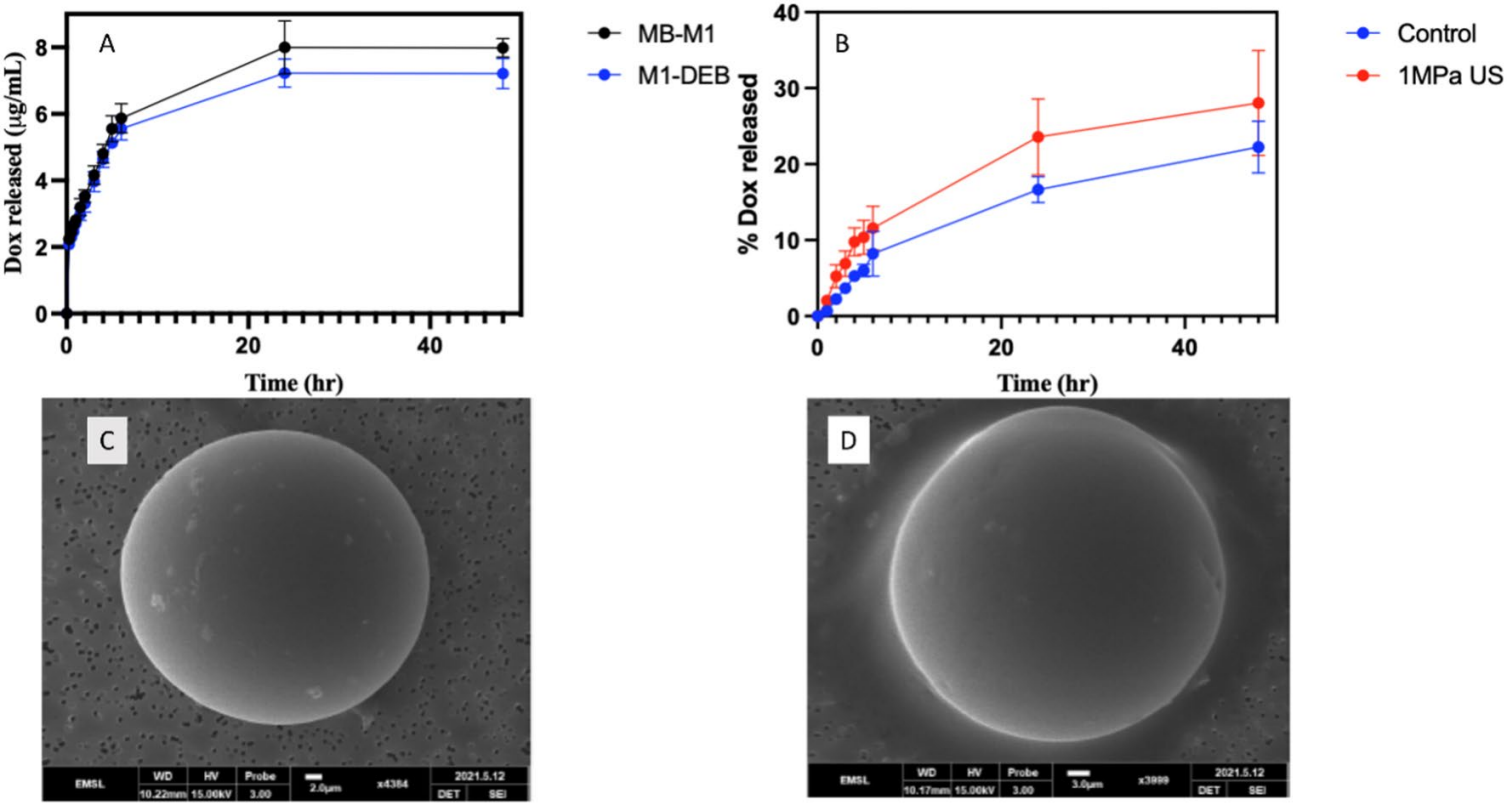
Release rate of doxorubicin from DEB and surface analysis of DEB surface, (**A**) comparison of release from M1 DEB and MB-M1 DEB loaded with Dox (100%) over 48 h, (**B**) Release from MB-M1 DEB loaded with 100% Dox with and without applied HIFU. (**C**) SEM image of MB-M1 without HIFU application and (**D**) SEM image of MB-M1 after HIFU application. For all graphs (n = 3).

A microbubble cavitating on a rigid DEB surface could cause pitting or cratering, potentially changing the release rate and quantity of Dox released from the DEB. The electrostatic attachment of MB to DEB could also impact release with or without ultrasound due to the charged lipids affecting the DEB surface properties. To investigate this, the release of Dox at 37 °C in saline was measured for 100% Dox loaded MB-M1 DEB and M1 DEB over 48 h. No difference in release was observed over time indicating that the MB had no impact on the release profile in the absence of HIFU (Fig. 6A). MB-M1 DEB with 75% Dox loading showed no change in the amount of Dox released with and without HIFU at each time point (Supplementary Information). However, at 100% Dox loading, a 5.8 ± 4.3% increase in the percentage of Dox released was observed after HIFU leading to more Dox being in solution at 24 h (p = 0.036). However, there was no difference in total Dox release after 48 h (p = 0.122) as shown in Fig. 6B. To investigate the faster release rate of Dox from the beads, the surface was explored by scanning electron microscopy (SEM). SEM indicated that microbubbles alone with or without HIFU had minimal impact on the bead surface indicating no damage was caused by the cavitating microbubbles (Fig. 6C and D). The faster release rate is not caused by damage to the bead surface but likely from an increasing flow around the DEB from cavitating microbubbles affecting local concentration gradients which could increase the release rate^47^. Another potential cause could be localized heating from the ultrasound on the beads resulting in a faster release rate. This could also be augmented by the localized heating from the cavitating microbubbles as well. The augmented release of Dox at 100% loading suggests that MB-M1 DEB could be used in conjunction with cavitation enhancement at the loading level used in patients.

### Dox distribution within a TMM

**Fig. 7:**
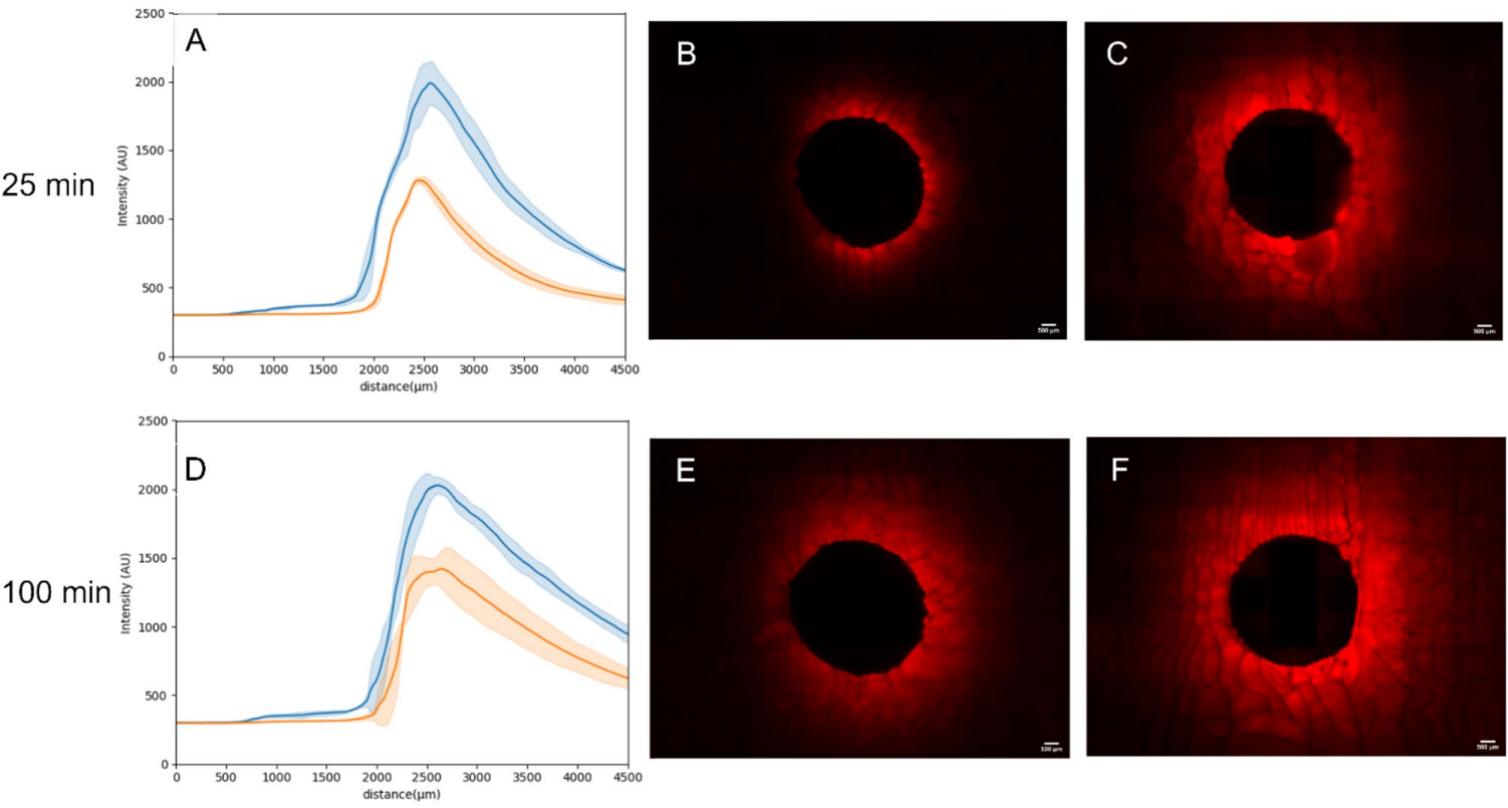
Delivery of Dox to a tissue mimicking material from Dox loaded MB-M1 DEB at 100% loading with (blue line) and without (red line) HIFU (1 MPa). (**A**) distribution of Dox around an agar vessel mimic with 100% Dox loaded MB-M1 DEB after 25 min with HIFU (Blue) and without HIFU (orange). Fluorescence imaging of the agar vessel mimic (**B**) without HIFU and (**C**) with HIFU after 25 min. (**D**), (**E**) and (**F**) show the parallel result after 100 min. For all graphs, n = 3. Scale bar is 100 µm in all images.

To investigate enhanced delivery into tissue, a TMM vessel mimic was developed from a 3D printed mold which could be adapted to have HIFU applied. The MB-M1DEB were injected into the channel which was large enough to be observed via MRI. The MRI was then used to target the HIFU to the channel along the phantom. The phantom was sectioned after HIFU application along the length of the channel or sham exposure. Analysis in FIJI using radial profiles, via a similar method to Mikhail et al.^25^ allowed visualization of the distribution of Dox around the channel. 75% Dox loaded MB-M1 DEB showed no difference in Dox delivery and distribution within the TMM with and without HIFU indicating that an increased quantity of MB does not have an impact on improving delivery relative to the quantity of Dox. (supplementary information). However, at 100% loading of Dox, but with a lower quantity of bound MB, HIFU caused a 60% increase in measured fluorescence intensity delivered to the vessel wall (p = 0.0013) compared to no HIFU and an increase in fluorescence at further distances from the vessel wall. At a distance of 2 mm the fluorescence intensity was 40% higher (p = 0.0004) than without HIFU (Fig. 7A–C). After 100 min, the increase was still greater than diffusion alone but was only 40% greater at the vessel wall (p = 0.0009) but remained 40% higher at a distance of 2 mm (p = 0.0037) as shown in Fig. 7D–F. These results show that MB cavitation on the DEB surface permeabilized the TMM and improved Dox delivery distribution into the phantom. This is likely due to the cavitation enhancing release from the DEB and permeability of the TMM and driving already released Dox into the vessel wall. This provides evidence that combined HIFU and MB-DEB can increase the amount of therapeutic delivered to tissue and increase the volume of tissue reached by the therapeutic. However, further studies are required to better characterize the significance of enhanced delivery and to link enhanced distribution and delivery to therapeutic efficacy. The HIFU conditions chosen to cause the enhanced delivery were based on previous literature with MB. These ultrasound conditions have not been optimized nor the exact method of enhancing drug permeation understood. It is likely a combination of microjetting, microstreaming and potentially bubble tunnelling, all resulting from MB cavitation. Different ultrasound conditions such as lower pressures to cause stable cavitation or a reduced duty cycle to limit heating will be explored in future work to optimize the MB-DEB response.

## Conclusion

Microbubbles were electrostatically bound to M1, M1-DEB and LUMI. The amount of microbubble binding was dependent upon Dox loading, but a significant number of microbubbles were still bound to the DEB surface at 100% Dox loading (37.5 mg Dox/mL microbeads). The MB-M1 DEB showed no structural damage from bubble attachment or cavitation of bound MBs. HIFU increased the rate of Dox release from 100% Dox loaded MB-M1 and increased the fluorescence intensity of Dox delivered into a TMM wall by 60% relative to sham HIFU exposure. HIFU also increased the penetration depth of Dox in the phantom at each point up to the limit of measurement at 2 mm with a 40% increase in fluorescence intensity. This scale of penetration compares favorably to reported temporospatial diffusion of Dox from DEB in vivo. These data support the concept of using MB to create localized cavitation and enhance drug delivery from DEB. Further investigation is needed to optimize the ultrasound exposure parameters. The MB-LUMI were able to be differentiated from LUMI alone using ultrasound imaging, providing for another method of assessing TACE. Experiments in an in vivo model are needed to support further translation toward clinical application. Penetration and temporospatial delivery of drug may be enhanced by rational mechanistic design of drug plus device methodologies to optimize local and regional therapies such as TACE and DEB-TACE.

## Limitations

This work explored the proof of concept for a combination of ultrasonic cavitation and drug eluting beads to enhance the delivery and distribution of dox. This has been shown in an in vitro TMM. However, the MB-DEB constructs have not been optimized and the in vitro models are a larger size than would be expected in vivo. The MB-DEB ratio has not been optimized and unbound cationic MB remain in solution. Unbound MB could potentially cause side effects or lead to an immune response which requires optimization of the binding process through bubble removal or another binding technique such as maleimide-thiol^48^. Sedimentation was also used for imaging rather than blockage of a vessel. No therapeutic efficacy has been observed. However, this study provides proof that HIFU can enhance the release rate and delivery from DEBs coated with microbubbles and that the concept can be taken forward for in vivo models.

## Future work and clinical translation

For this proof-of-concept study, optimizing the MB-DEB was not explored. Future work will focus on treatment of an in vivo tumor model using HIFU and MB-DEBs. The studies will also explore safety related to the potential clustering of beads with attached microbubbles. Removal of unbound MB will be investigated to prevent any untoward effects on ultrasound imaging and drug delivery. This will likely be through a combination of controlling the quantity of MB added or subsequent removal through flotation or centrifugation. Enhanced release and distribution will be studied, coupled to therapeutic efficacy. Study of the durability of the MB-DEB required for delivery, i.e., transcatheter infusion, and injection parameters will be essential.

## Supplementary Information

Supplementary Figures.

## Data Availability

The authors confirm that the data supporting the findings of this study are available within the article and its supplementary materials. Additional supporting materials available upon reasonable request.
