# Supplementary material for: Microbubbles bound to drug-eluting beads enable ultrasound imaging and enhanced delivery of therapeutics
Source: Sci Rep. 2024 Sep 9;14:20929. doi: 10.1038/s41598-024-71831-3 (PMC11383944; doi:10.1038/s41598-024-71831-3)
Supplement: Supplementary file 1 — Supplementary Figures. [file 41598_2024_71831_MOESM1_ESM.docx]

## Supplementary Figures


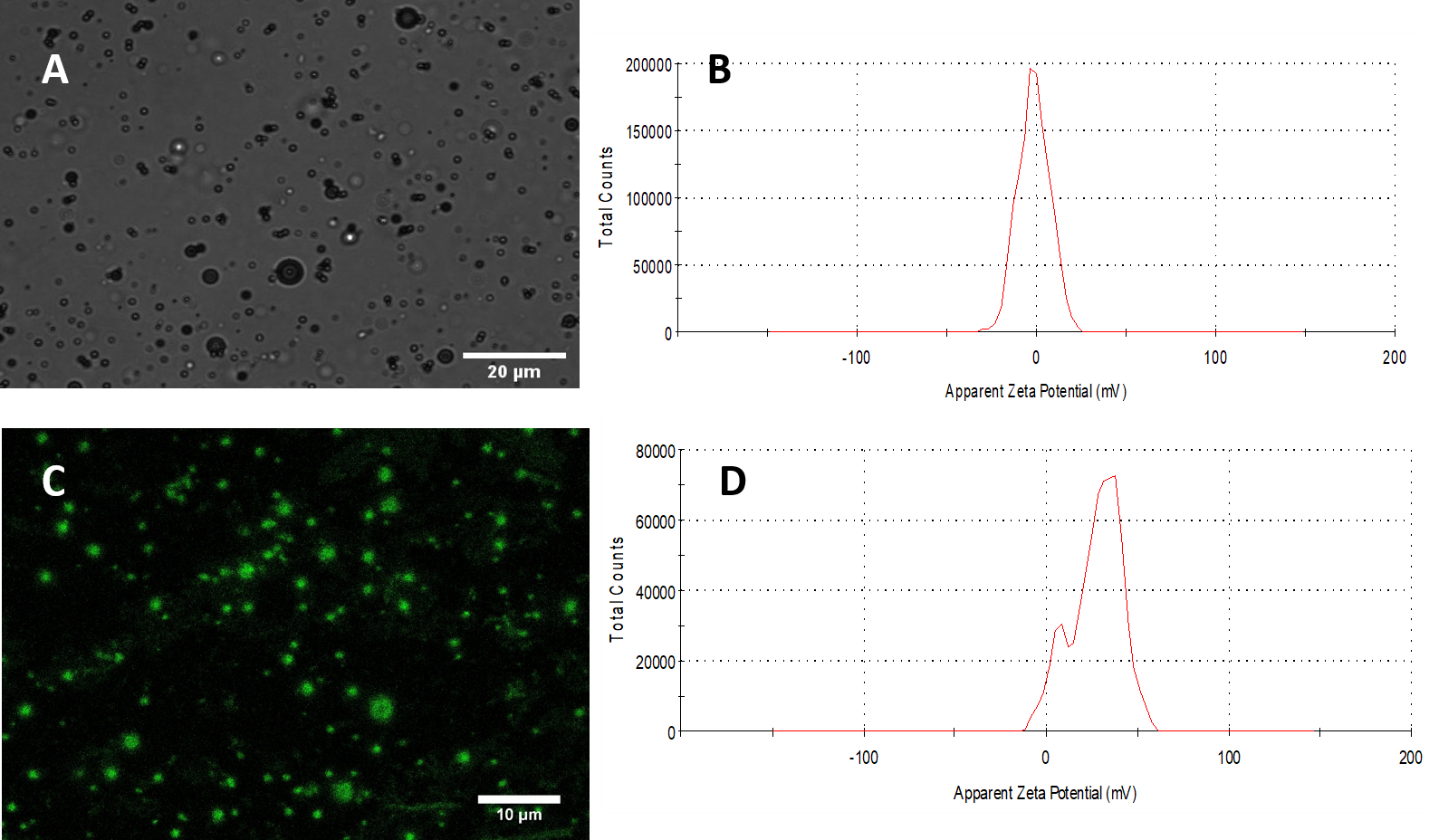


Figure 1, Characterization of microbubbles, A) Image of neutral MBs, B) zeta potential of neutral MBs, C) fluorescence microscopy image of cationic MBs with the fluorophore NBD embedded in the lipid shell and D) zeta potential of cationic MBs.


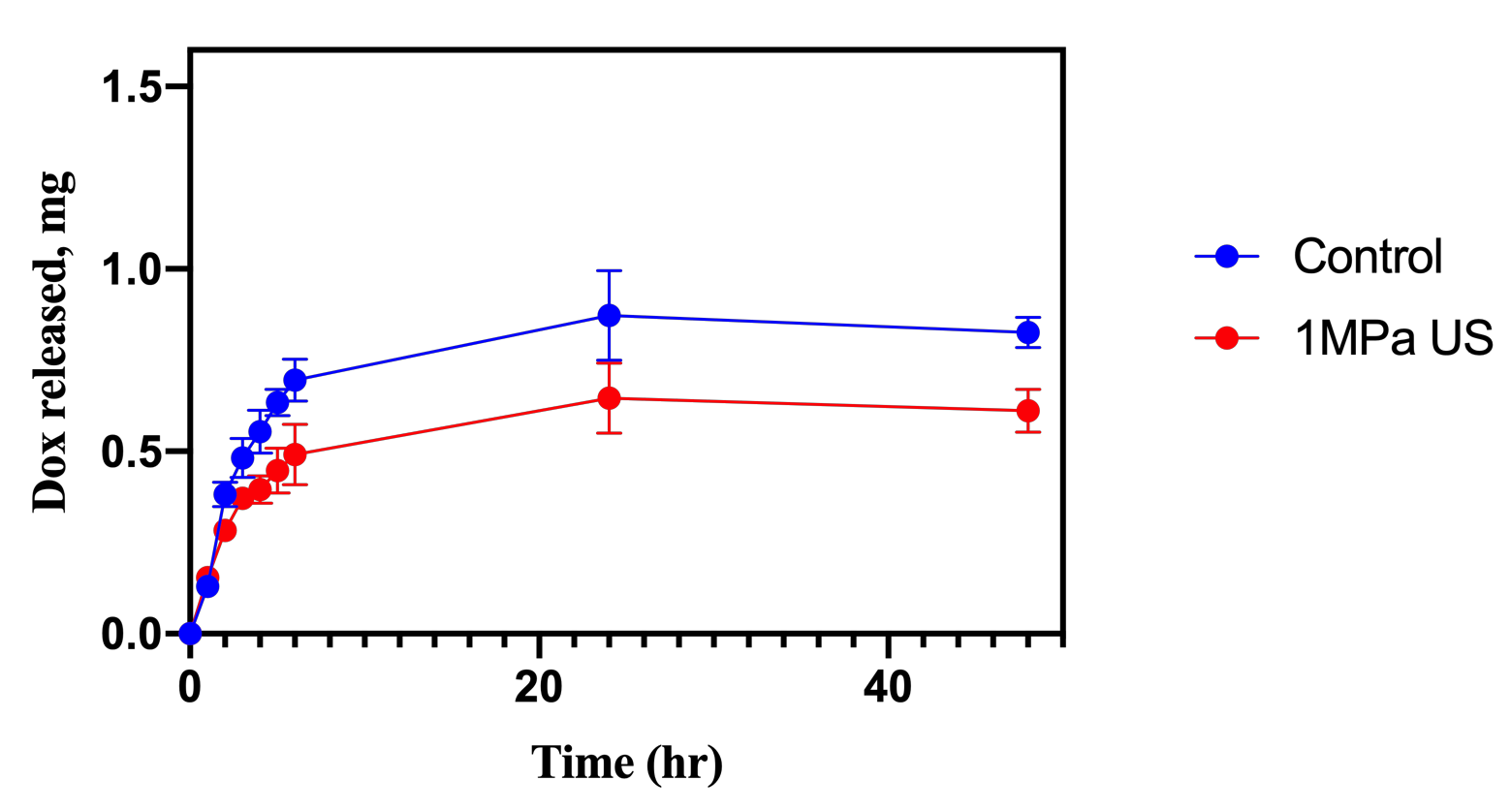


Figure 2, Dox release rate of 75 % Dox loaded beads with and without 1 MPa of ultrasound.


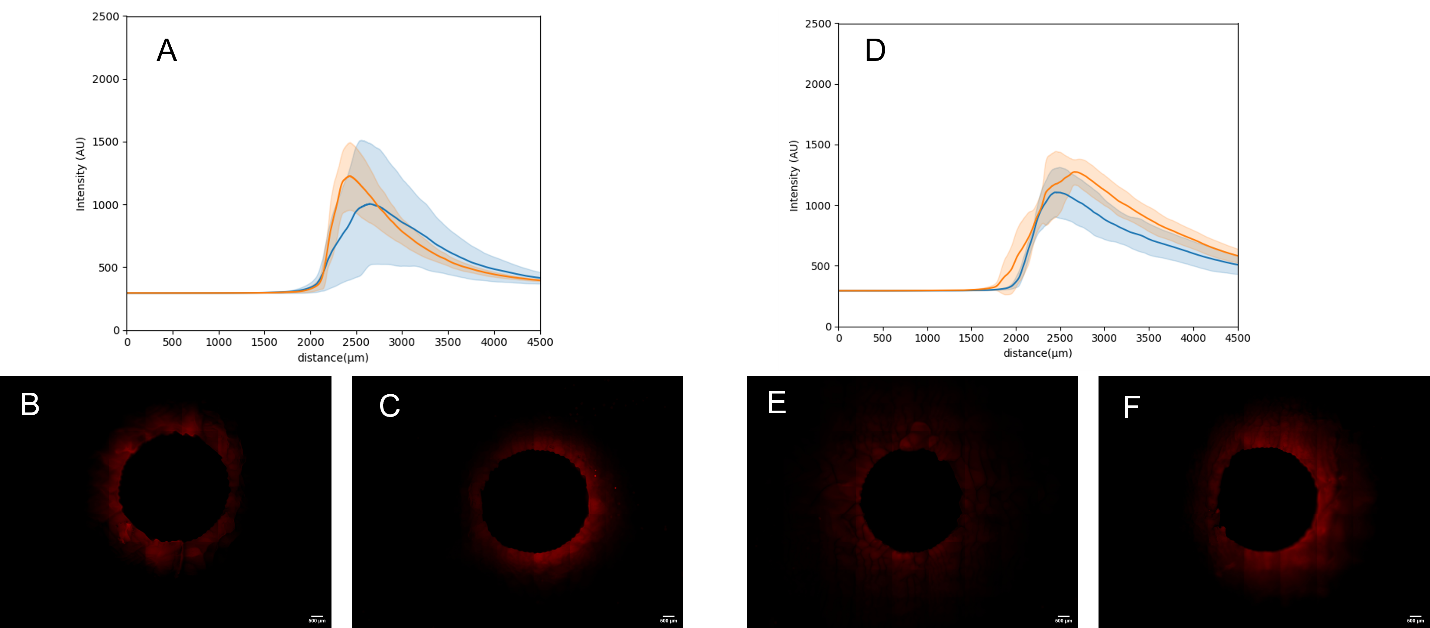


Figure 3, Delivery of Dox to a TMM from Dox loaded DEB-MB at 75 % loading with and without HIFU (1 MPa). A) distribution after 25 min of Dox around an agar vessel mimic with HIFU (Blue) and without (red) and examples of vessel B) without HIFU and C) with HIFU. D), E) and F) show the result 100 min later. (n =3 for all graphs). Scale bar is 100 µm in all images.
